# Regulation of GAD65 expression by SMAR1 and p53 upon Streptozotocin treatment

**DOI:** 10.1186/1471-2199-13-28

**Published:** 2012-09-14

**Authors:** Sandeep Singh, Varsheish Raina, Pavithra Lakshminarsimhan Chavali, Taronish Dubash, Sreenath Kadreppa, Pradeep Parab, Samit Chattopadhyay

**Affiliations:** 1 Samit Chattopadhyay, PhD, Scientist-G, National Centre for Cell Sciences, Pune, 411007, India; 2 Sandeep Singh, Assistant Professor, Centre for Human Genetics, School of Health Sciences, Central University of Punjab, Bathinda, 151001, India

**Keywords:** SMAR1, Diabetes, GAD65, p53, Streptozotocin

## Abstract

**Background:**

GAD65 (Glutamic acid decarboxylase 65 KDa isoform) is one of the most important auto-antigens involved in Type 1 diabetes induction. Although it serves as one of the first injury markers of β-islets, the mechanisms governing GAD65 expression remain poorly understood. Since the regulation of GAD65 is crucial for the proper functioning of insulin secreting cells, we investigated the stress induced regulation of GAD65 transcription.

**Results:**

The present study shows that SMAR1 regulates GAD65 expression at the transcription level. Using a novel protein-DNA pull-down assay, we show that SMAR1 binding is very specific to GAD65 promoter but not to the other isoform, GAD67. We show that Streptozotocin (STZ) mediated DNA damage leads to upregulation of SMAR1 and p53 expression, resulting in elevated levels of GAD65, in both cell lines as well as mouse β-islets. SMAR1 and p53 act synergistically to up-regulate GAD65 expression upon STZ treatment.

**Conclusion:**

We propose a novel mechanism of GAD65 regulation by synergistic activities of SMAR1 and p53.

## Background

Type1 diabetes is an immunologically encountered autoimmune disease characterized by specific destruction of beta cells of islets of langerhans residing in pancreas [1]. This specific response to beta cells is caused due to immune response against self molecules that behave as non-self (known as auto antigens). GAD (Glutamic acid decarboxylase) which is involved in synthesis of Gamma-Amino butyric acid or GABA, a neurotransmitter inhibitor [2,3], is considered to be one of the strongest candidate auto antigens involved in triggering beta-cell-specific autoimmunity [4]. Majority of recent type 1 diabetes patients and pre-diabetic subjects have anti-GAD antibodies in their sera, as do non-obese diabetic (NOD) mice, one of the best animal models for human type I diabetes. Immunization of young NOD mice with GAD results in prevention or delay of the disease as a result of tolerizing auto reactive T cells [4]. On the other hand over-expression of GAD65 transgene in animal tissues can exacerbate the disease instead of tolerizing the animal [4]. GAD usually occurs in two major isoforms; GAD65 and GAD67, encoded by two non-allelic genes located on different chromosomes [5] and are differentially regulated in various mammalian species. In beta cells of human and rat GAD65 is predominant, while in mouse though both the forms occur GAD67 predominates over GAD65 [6-8]. Although the expression level of GAD65 is extremely low in mouse β-islets, it is one of the major islet auto antigens [7-9]. Despite studies on the role of GAD65 in diabetes induction using various in-vitro and in-vivo models of spontaneous autoimmune diabetes [10,11], the initial islet-specific factor(s) and the molecular mechanisms triggering the aberrant expression of GAD65 is still not very clear. The smaller form of the GAD auto-antigen namely GAD65, is a major target of humoral autoimmunity in type-1 diabetes. Recent data suggest that GAD65 expression in beta cells varies according to the functional state and the kind of stress [12]. High glucose and glutamine have been shown to be putative positive regulators of GAD65 expression [13-15], while cytokines like IL-1 beta have been shown to act as negative regulators [15]. GAD is an enzyme found in high concentration in the pancreas and brain where it catalyzes the conversion of glutamic acid to gamma-amino butyric acid (GABA). GABA is an inhibitory neurotransmitter that is important in the pancreas as a messenger between neurons and pancreatic cells [16,17]. Specific experiments in animal models have shown that GAD expression is necessary for the autoimmune destruction of cells. One study in particular, performed on NOD mice (which are close to humans in their autoimmune manifestation of IDDM), incorporated an antisense GAD transgene into a subset of mice and found that IDDM development was significantly reduced [18].

Streptozotocin (STZ), derived from a fermentation broth of *Streptomyces achromogenes*, has been widely used to study the beta cell destruction both in-vitro and in-vivo models [19,20]. The drug Streptozotocin (STZ) is a glucose analogue (N-[methylnitrosocarbamoyl]-D-glucosamine) that is rapidly transported into the β-cells via the glucose transporter, Glut2 [21] and is known to be metabolized readily upon entry into the cell. The exact mechanism of STZ’s toxicity is not fully understood, but it has been suggested that its primary effect on beta cells is DNA damage by alkylation, unscheduled DNA synthesis, DNA adducts, chromosomal aberrations and DNA strand breaks induced by free radical generation [22,23].

The tumor suppressor protein p53 can cause cell cycle arrest upon DNA damage induced activation [24-30]. In doing so, it facilitates the repair of damaged DNA or eliminates irreversibly damaged or abnormally growing cells to prevent potential transformation. Mdm2 helps to maintain steady state levels of p53 under normal condition through ubiquitination pathway [31-33]. STZ has also been shown to induce p53 protein in MIN6 cells [34] but the exact mechanism is largely unknown. One study also reported p53 antibodies circulating in patients suffering from type-1 diabetes [35].

Gene regulation is one of the most complex processes involving cross talk between a variety of proteins, nuclear matrix, DNA and many other DNA binding factors. The function of nuclear matrix is to provide a solid platform for efficient transcription. A number of matrix associated DNA region binding proteins (MARBPs) are known to be involved in regulation of transcription. SMAR1 is one such MARBP which was earlier shown to be involved in the regulation of Cyclin D1 and CK8 expression [36,37]. SMAR1 specifically binds to putative MAR (MARβ) of the transcriptional enhancer (Eβ) at the T-cell receptor-β locus [38,39]. This protein is known to cause cell cycle arrest and activates p53 through its serine 15 phosphorylation as well as through disruption of its interaction with MDM2 [40-43]. It is a potent tumor suppressor protein and significantly downregulated in higher grades of breast cancer [44].

The present study delineates a novel mechanism of regulation of GAD65 expression by two tumor suppressor proteins, SMAR1 and p53. We show that both the proteins can individually and synergistically upregulate GAD65 expression. We demonstrate that SMAR1 binds to GAD65 promoter in vitro and in-vivo to upregulate its mRNA expression. Our results show that STZ treatment leads to upregulation of SMAR1 and p53 expression. On the other hand MDM2 expression is downregulated leading to increased stability of p53. The stabilized p53 in turn binds to SMAR1 leading to an increased expression of GAD65. We also show a temporal increase of SMAR1 and p53 proteins on GAD65 promoter upon STZ treatment. Further studies using mouse β-islets confirmed our findings regarding the synergy between p53 and SMAR1 in GAD65 expression. Taken together, our results reveal a novel mechanism of regulation of major auto antigen GAD65 by SMAR1 and p53.

## Results

### SMAR1 binds to multiple DNA sequences in genome

**Figure 1:**
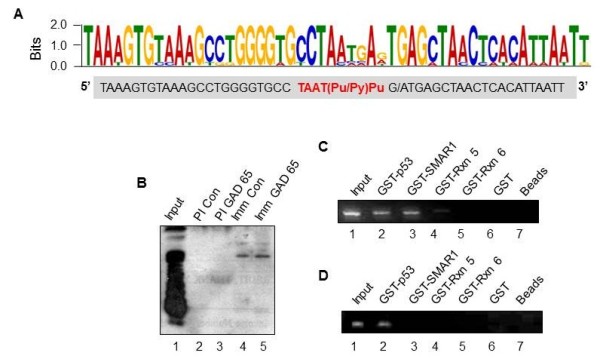
**Isolation of DNA fragments bound by SMAR1. A**. MEME software based alignment of the SMAR1 binding DNA sequences for the consensus for SMAR1 binding. **B**. CNBr pull down assay (by consensus and by GAD65 promoter DNA) samples were processed for western blot analysis using SMAR1 specific antibody. PI represents pre-immune and Imm represents the immune complexes. **C** &**D**. GST-p53, GST-SMAR1, GST-Rxn5 (DNA binding domain of SMAR1), GST-Rxn6 (Protein binding domain of SMAR1) along with GST and bead only controls was used for in-vitro pull down assays for DNA-protein interactions. The resulting DNA fragments were amplified by PCR using GAD65 (C) and GAD67 (D) specific primers.

SMAR1 is documented to be a transcriptional activator/repressor in a context dependent manner, based on its DNA binding abilities. In vivo, SMAR1 binds to the promoters of Cyclin D1 and 5' UTR of cytokeratin 8 [36,37]. To gain a better understanding of the transcription regulatory activities of SMAR1, we wanted to delineate various genomic DNA sequences bound by SMAR1. Therefore, we resorted to a novel pull down assay. Briefly, mouse genomic DNA was sheared to obtain small fragments of approximately 500 bp. These fragments were incubated with glutathione bead bound GST-SMAR1 recombinant protein and the bound DNA fragments were subsequently sequenced. Glutathione beads as well as bead bound GST were the controls used to negate non- specific binders. The resulting fragments were sequenced and then aligned using MEME software and a 50 mer consensus was derived (Figure 1A). Further manual alignments of the derived sequences lead to identification of a hexamer (TAATPu/Py Pu) consensus SMAR1 binding sequences where first four nucleotides are conserved while fifth one can be either pyrimidine or purine and the last nucleotide is either of the purines (Figure 1A, sequence shown in red). Further, we were able to validate our method by identification of some of the already known promoters bound by SMAR1, including cyclin D1 [36], Bax [45], 5' UTR of cytokeratin 8 [37]. One of the unique promoters found to be bound by SMAR1 was GAD65 while the other isoform of GAD i.e. GAD67 promoter did not show any binding. In order to confirm that SMAR1 indeed binds to the GAD65 promoter, we performed CNBr pull down assay. The consensus sequence from GAD65 promoter and the general consensus oligonucleotide were individually coupled to CNBr beads and Rin 5f cell lysate was passed through the column. The protein samples were eluted from the CNBr columns and processed for Western blotting using SMAR1 specific antibody. Our result showed that SMAR1 bound with equal propensity to both GAD65 promoter sequence and the general consensus oligo (Figure 1B, lanes 4 and 5). In-vitro pull down assays followed by PCR amplification showed that GST-p53, 350–548 aa (DNA binding domain of SMAR1, 36) and the full-length SMAR1 were able to precipitate GAD65 promoter (Figure 1C, lanes 2, 3 & 4). The protein binding domain of SMAR1 i.e. GST-SMAR1 (160–350 aa), GST bound beads and glutathione beads alone did not show any amplification (Figure 1C, lanes 5–7) showing the specificity of the interaction. All these samples were further analyzed for GAD67 promoter binding. While we detected GAD67 promoter bound to GST-p53, neither the full length nor the DNA binding domain of SMAR1 showed any binding to GAD67 promoter (Figure 1D, lane 2). It is well known that p53 binds to GAD67 promoter [34]. From the above mentioned results, it is clear that SMAR1 specifically binds to the consensus sequence in GAD65 promoter.

GAD65 has a TATA less promoter and various other factors are known to regulate it. Therefore we analyzed GAD65 promoter for other transcription factor binding sites *in silico.* A careful analysis of the sequence showed that SMAR1 binds 870 bp upstream of transcription start site. We found a strong p300 consensus element (~820 bp upstream) and a p53 binding site (~560 bp) juxtaposed to SMAR1 binding sites. A detailed map of various binding sites is shown in Additional file 1.

### SMAR1 binds to GAD65 promoter and upregulates its expression

**Figure 2:**
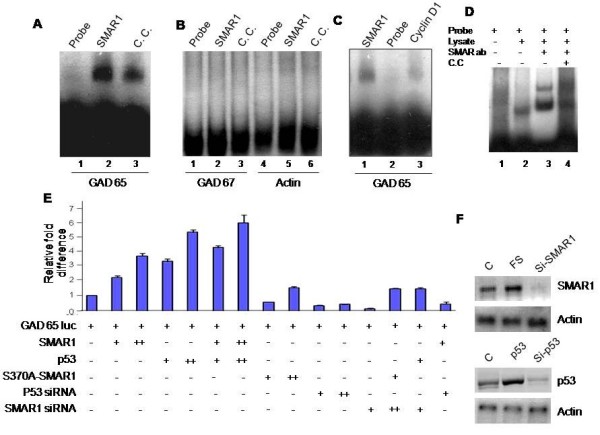
**SMAR1 binds to GAD65 promoter. A**. Electro mobility Shift assay (EMSA) was done using GAD65 promoter DNA fragment which is bound by GST-SMAR1 in EMSA (lane 2) while cold competitor (C.C.; lane 3) reduced the binding showing the specificity of the interaction. **B**. Promoter fragments of GAD67 and actin was used as negative controls and did not show any binding with GST-SMAR1 proteins. **C**. Competitive EMSA shows SMAR1 interaction with GAD65 promoter (lane 3) is greatly reduced in presence of a competitor of cyclin D1 promoter oligo compared to no competition lane (lane 1). **D**. Supershift assay using Rin5f cell lysate without (lane 2) or with SMAR1 antibody (lane 3) showing shift in the GAD65 promoter band. The upper band in lane 3 represents GAD65-SMAR1-SMAR1 ab complex while lower band shows SMAR1-GAD65 promoter complex only. **E**. Luciferase assay showing that SMAR1 and p53 together leads to up-regulation of GAD65 promoter activity. Coexpression and knock down of SMAR1 and p53 in various combinations was used to look of luciferase activity of GAD65 promoter. **F**. Rin 5f cells were treated with FLAG-SMAR1 as well as siRNA of SMAR1 and western blot was carried out to test their effect over SMAR1 expression (upper panel). Similarly cells were treated with FLAG-p53 as well as siRNA of p53 and western blot using p53 antibody was done to check its expression levels (lower panel).

We further verified the binding of SMAR1 to GAD65 promoter using mobility shift assays. A 120 bp probe from GAD65 promoter which harbors the potential MAR and SMAR1 consensus binding site was radiolabelled and used for the assays. EMSA using radiolabelled GAD65 promoter probe showed a SMAR1-DNA complex formation (Figure 2A, lane 2) and a cold competitor reduced this complex formation (Figure 2A, lane 3) showing the specificity of binding. GAD67 and Actin (Figure 2B lanes 1–3 and 4–6 respectively) promoter specific probes did not show any complex formation with SMAR1 recombinant protein. Also, competition with cyclin D1 promoter oligo, greatly reduced the complex formation on GAD65 oligo and reflected the specificity of the complex formation (Figure 2C lane 3). Similarly, super-shift assays with SMAR1 specific antibody on using Rin cell lysate helped document SMAR1 complex formation on GAD65 promoter oligo (Figure 2D lane 2 and 3). The use of cold competitor in this experiment significantly reduced the specific complex formation (Figure 2D, lane 4).

After confirming that SMAR1 binds to GAD65 promoter, we proceeded to check the in vivo effect of SMAR1 binding on the promoter. It is known that GAD65 is the predominant form in rat while in mouse both the forms are expressed. Rat insuloma cell line Rin 5f cells were co-transfected with a luciferase reporter construct driven by GAD65 promoter, and expression plasmids/siRNAs of SMAR1 and p53. The results show that GAD65 promoter drives the expression of reporter gene upon over-expression of SMAR1 or p53 witnessed by an increase of ~ 4 and ~ 4.5 folds respectively (Figure 2E). On the other hand, knock-down of either of these proteins leads to a decreased luciferase activity driven by GAD65 promoter. Over-expression of SMAR1 and p53 together lead to the highest luciferase counts (~ 6 folds increase) indicating their additive effect on GAD65 promoter. On the other hand the knockdown of both lead to negligible promoter activity. Knock-down of p53 and over expression of SMAR1 partially rescued (~ 1.5 folds) the luciferase activity. These results indicate that although SMAR1 or p53 individually can up-regulate GAD65 promoter activity, their synergistic activity is required for maximal promoter activity that in turn reflects the transcriptional activation. On the other hand, either one of them is indispensible for activation of GAD65 promoter. It has been reported that phosphorylation of SMAR1 at serine 370 residue reduces its DNA binding activity [30; unpublished data]. Transfection of S370A mutant-SMAR1 led to a reduced GAD65 promoter activity compared to the wild-type SMAR1. This was not overcome by ectopic expression of p53 (Figure 2E, lanes 8 & 9 respectively). This result clearly indicates that direct binding of SMAR1 is essential for GAD65 promoter activation and that the effect of SMAR1 is not through stabilization/activation of p53. In order to verify our results we performed western blot analysis to confirm over expression as well as siRNA mediated knockdown of SMAR1 and p53. Figure 2F shows the expression levels of SMAR1 (upper panel) as well as p53 (lower panel) in Rin5f cells.

### SMAR1 leads to upregulation of GAD65 expression

**Figure 3:**
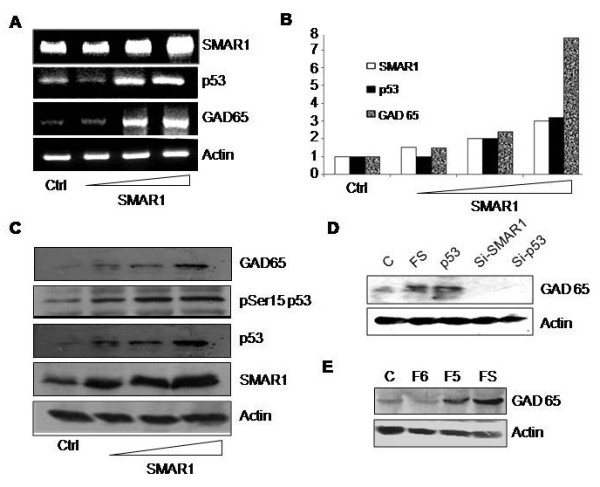
**SMAR1 upregulates GAD65 expression. A**. RT-PCT analysis of GAD65 and p53 upon dose dependent SMAR1 over-expression. Actin was used as loading control. **B**. Densitometric analysis of the RT-PCR showing ~4 fold increase in p53 and ~7 fold increase in GAD65 mRNA levels upon SMAR1 over-expression. **C**. SMAR1 was over-expressed in Rin5f cells and samples were processed for western blot analysis 48 hrs post transfection. Figure shows western blot analysis of these samples using GAD65, phospho serine 15 p53 and SMAR1 expression while actin was used as loading control. **D**. SMAR1 and p53 over expression leads to up-regulation (lanes 2 & 3) while their knock-down abolish the basal expression of GAD65 (lanes 4 & 5). **E**. DNA binding domain (F5), protein interaction domain (F6) of SMAR1 and full length SMAR1 (FS) transfections led to increased expression of GAD65.

Next we verified the expression of GAD65 upon over expression of SMAR1. RT-PCR results showed that upon SMAR1 over expression, GAD65 and p53 mRNA levels are elevated in a dose dependent manner (Figure 3A). Densitometric analysis of the RT-PCR showed that upon transfection of 2 μg of SMAR1, GAD65 mRNA levels were 7 folds higher compared to the control cells, while p53 mRNA levels were increased by 3 folds (Figure 3B). Similarly, western blot analysis of the same showed increased GAD65 protein levels in a dose dependent over-expression of SMAR1. Earlier it has been reported that SMAR1 leads to phosphorylation of p53 at serine 15 and subsequent stabilization [41-43]. We also observed increased serine 15 phosphorylation as well as total p53 levels (Figure 3C). Actin was used as loading control. Also, our results showed that over expression of either of SMAR1 or p53 lead to elevated GAD65 protein levels while their knockdown decreased GAD65 expression (Figure 3D). We then transfected different truncations of SMAR1 pertaining to DNA binding (350–548 aa; F5) and protein interacting domain (160–350 aa; F6) of SMAR1 along with the full length SMAR1. Our results showed that full length as well as DNA binding domain of SMAR1 lead to increased expression of GAD65 (Figure 3E). Thus, our results show that DNA binding activity of SMAR1 is necessary for upregulation of GAD65 expression, while the protein interacting domain which has been shown to phosphorylate and activate p53 is not sufficient to drive p53 dependent expression of GAD65.

### STZ induced DNA damage leads to upregulation of GAD65, SMAR1 and p53

**Figure 4:**
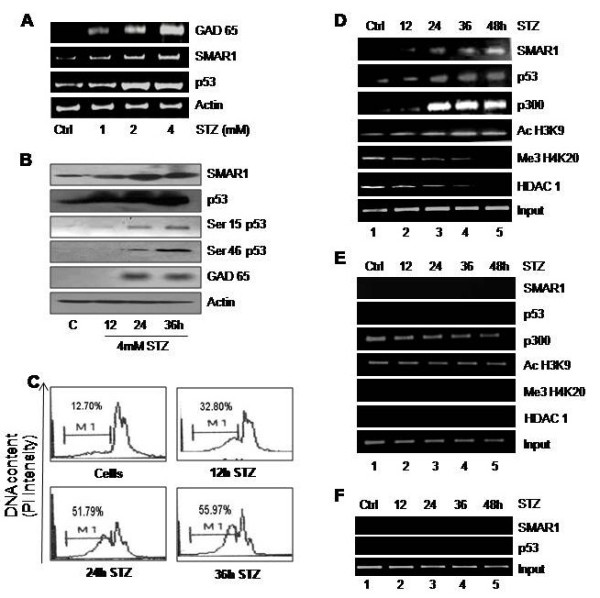
**STZ treatment leads to upregulation of SMAR1, p53 and GAD65 expression. A**. RIN cells were treated with STZ in a dose dependent manner as indicated for 24 hrs followed by RT-PCR analysis of SMAR1, p53 and GAD65 mRNA expression. **B**. Western blot analysis of SMAR1, ser46 p53, ser15 p53, total p53 and GAD65 expression upon 4 mM STZ treatment in a time dependent manner. **C**. Same cells treated with 4 mM STZ were used for FACS analysis at indicated time points. **D**. RIN cells were treated with 4 mM of STZ and cells were harvested after indicated time points. The samples were than processed for ChIP analysis of GAD65 promoter upon STZ treatment for binding of p53, SMAR1, p300 along with various chromatin activation and repression markers. **E** &**F**. ChIP analysis of upstream promoter region of GAD65 (**E**) and actin (**F**) which were used as negative controls did not show any binding by SMAR1 or p53. The ChIP markers used represent activation (p300 & Ac H3K9) as well as repression (Me3H4K20 & HDAC1) of transcription.

It is known that GAD67 and p53 expression increases upon STZ treatment [34], which prompted us to verify the effect of STZ on SMAR1. As reported earlier, SMAR1 is a DNA damage responsive protein and is upregulated upon DNA damage induced by H_2_O_2_, γ-irradiation, Camptothecin and Doxorubicin. Our results here show that STZ treatment to Rin cells led to elevated mRNA levels of SMAR1, p53 and GAD65 in a dose dependent manner (Figure 4A). Western blotting of the same showed that SMAR1, p53 and GAD65 protein levels were elevated upon 4 mM STZ treatment. Interestingly Serine 15 as well as ser 46 phosphorylation of p53 also increases, indicating that prolonged STZ treatment leads to apoptosis through serine 46 phosphorylation of p53 (Figure 4B). FACS analysis of the cells also showed increased number of apoptotic cells upon STZ treatment (Figure 4C). Therefore, we examined the kinetics of SMAR1 and p53 binding to GAD65 promoter upon STZ treatment. Rin cells were treated with STZ for indicated time points and processed for ChIP analysis. Our results show that there is increased binding of SMAR1 as well as p53 on GAD65 promoter in a time dependent manner upon STZ treatment (Figure 4D). Histones on GAD65 promoter were hyper-acetylated in response to STZ treatment showing chromatin activation. The increase in recruitment of p300 to GAD65 promoter indicates chromatin activation that is reflected in increased GAD65 expression. On the other hand HDAC1 as well as Me3H4K20 showed time dependent loss of binding to GAD65 promoter (Figure 4E). HDAC1 recruitment causes local deacetylation of the chromatin thus leading to repressed state. Tri methylation of histone 4 lysine 20 residue is also indicative of the compact/repressed state of chromatin. All these results indicate that upon STZ treatment, SMAR1 and p53 binds to GAD65 promoter and recruit p300 which then leads to activation of GAD65 promoter. Upstream promoter region of GAD65 and actin promoter served as negative controls (Figure 4E, F).

### Effect of STZ treatment on mouse β-islets

**Figure 5:**
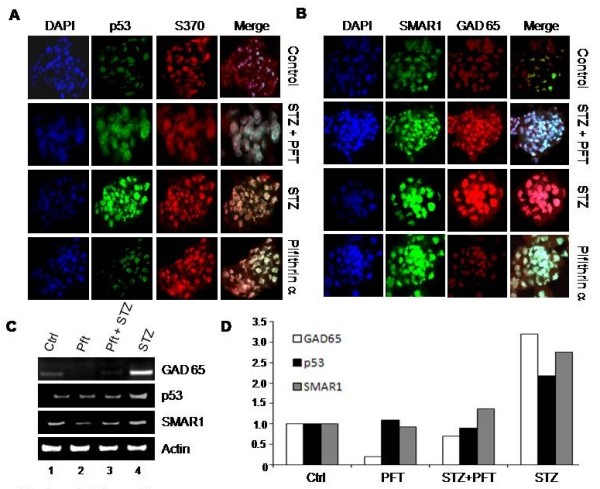
**STZ treatment in mouse β-islets leads to upregulation of SMAR1, p53 and GAD65 expression. A** &**B**. β-islets were treated with STZ and PFT individually as well as in combination followed by fluorescent staining using S370-SMAR1 (phosphor SMAR1 at S370 locus), total SMAR1, P53 and GAD65 antibodies. DAPI was used as nuclear marker to distinguish between cytoplasm and the nucleus. **C**. RT-PCR analysis of SMAR1, p53 and GAD65 upon streptozotocin (STZ), pifithrin-α (PFT) and PFT + STZ treatments. Actin was used as internal loading control. **D**. The same RT-PCR data was used for densitometric analysis. The graph indicates the respective expression of GAD65, p53 and SMAR1 expression at different treatments while the expression in control cells is taken as 1. Thus these values indicate expression comparison of a single molecule with different treatments and cannot be used to compare expression of two different genes.

After verifying that SMAR1 leads to increased expression of GAD65 upon STZ induced DNA damage, we wanted to verify our results in-vivo. Mouse β-islets were isolated using collagen as detailed in materials and methods section and were cultured for 6 hrs prior to treatment. The islets were then treated with high (33 mM) and low (7 mM) dose of glucose, p53 activity inhibitor Pifithrin-α PFT and STZ (4 mM) for 24 hrs followed by confocal as well as RT-PCR analysis. Confocal results showed that STZ treatment leads to increased expression of SMAR1, p53 as well as GAD65 (Figure 5A & B). Phosphorylation of SMAR1 at serine 370 residue is also increased upon STZ treatment (Figure 5A). This post-translationally modified form of SMAR1 is known to have higher affinity for DNA [30]. On the other hand the islets treated with PFT along with STZ did not show much change in GAD65 expression indicating that p53 is required for its higher expression.

RT-PCR in the islets showed that treatment with high glucose or STZ lead to increased expression of SMAR1, GAD65 and p53, while mdm2 mRNA levels are reduced (Figure 5C). Actin was used as loading control. On the other hand, islets treated with both p53 inhibitor PFT and STZ showed only a marginal increase in GAD65 expression indicating that although SMAR1 alone can up-regulate the GAD65 expression, the combinatorial effect of SMAR1 and p53 is needed for sustained levels of GAD65 (Figure 5C, lane 3). The densitometric analysis of RT-PCR shows that upon STZ treatment, p53, SMAR1 and GAD65 mRNA levels increase by ~2.1; ~2.7 & ~3.2 folds respectively (Figure 5D). This increased GAD65 is secreted in the circulation and is considered as the first injury marker for β-islet injury [37].

## Discussion

Transcription factors play an important role in regulation of gene expression through their DNA binding properties. In order to understand the different functions of a transcription factor, elucidation of its target genes is of utmost importance. As discussed earlier, SMAR1 is a transcription factor and is involved in various cellular pathways through regulation of target gene expression like Cyclin D1 [36], CK8 [37] and Bax [46]. In order to further delineate various promoters bound by SMAR1, we employed a novel in vitro binding assay using recombinant GST-SMAR1 protein. Our protocol involved in vitro binding of recombinant protein with sonicated mouse genomic DNA fragments. The high salt buffer maintains a stringent condition for protein-DNA interaction. Subsequent cloning and sequencing of pulled DNA fragments lead to validation of known SMAR1 target promoters as well as elucidation of many new promoter fragments, GAD65 being one of them. The interesting aspect of this interaction was that SMAR1 was specifically binding to only GAD65 and not to the promoter of other isoform GAD67. Since GAD65 is a crucial protein in the induction of diabetes, we investigated the mechanism of its regulation by SMAR1. The major phenotype in type-1 diabetes is apoptosis, resulting in a progressive loss of pancreatic beta cells through autoimmune attack. On the other hand it has been shown that over-expression of GAD65 transgene in animal tissues can exacerbate the disease instead of tolerizing the animal though the exact mechanism remains unclear [4]. Another study using fetal mouse tissue, showed upregulation of GAD in pancreatic islet cells. This upregulation was shown to be caused by an impairment of mitochondrial complex I as a result of IDDM. Impairment of mitochondria resulted in an accumulation of glutamate that directly induced the upregulation of GAD in the cells [45]. It is speculated that GAD expression on pancreatic beta cells may be involved in the modulation of beta cell specific autoimmune response and/or modulation of the functional state of beta cell [47]. Much of the accumulated data from Type-1 animal models has come from the discovery of a novel diabetogenic compound Streptozotocin (STZ). The influence of STZ on the modulation of GAD expression has been shown earlier on GAD67 in MIN cells and also the INS cells treated with STZ show increased release of GABA in the culture medium that indirectly measures the rate of GAD expression [34,48]. Several key transcriptional factors like NFκB and p53 have been shown to regulate the expression of GAD. Although it has been shown that STZ can regulate GAD67 but its effect on GAD65 expression has not been precisely evaluated [34].

In the present study we for the first time show that SMAR1 binds to GAD65 promoter but not GAD67. Using a novel in-vitro pull down assay we derived a consensus DNA binding site for SMAR1. Bioinformatics analysis of various known SMAR1 binding sequences revealed that there is significant similarity between DNA sequences where SMAR1 binds. For example, Cyclin D1 and GAD65 share the identical hexamer sequence in their promoter where SMAR1 binds. CNBr pull down assay followed by western blotting (South-western blotting) confirmed that SMAR1 binds to the consensus as well as GAD65 promoter. Binding of SMAR1 to GAD65 promoter was further verified using EMSA. Our results suggest that SMAR1 and p53 binds to GAD65 promoter and synergistically regulate its expression. Transient transfections of SMAR1 or p53 followed by RT-PCR as well as luciferase assays showed that SMAR1 as well as p53 individually can up-regulate GAD65 expression. On the other hand whenever transfected together, the GAD65 expression increased many fold, indicating that both SMAR1 and p53 act cumulatively leading to an increased expression of GAD65. Our results indicate that DNA binding domain binds to GAD65 promoter while protein interacting domain might recruit various other transcription factors leading to upregulation of GAD65 promoter.

In our model also we have investigated the possible role of STZ in modulation of GAD65 expression through co-ordinated interaction of p53 and SMAR1. We first examined the effect of STZ on the expression of GAD65, p53 and SMAR1 in rat beta cell line, RINm5f. We found that STZ significantly enhanced the expression of p53, SMAR1 and GAD65 molecules both at mRNA and protein level. This increased expression was observed in a time and dose dependent manner STZ treatment also led to increased phosphorylation of p53 at serine 15 as well as serine 46. Serine 15 phosphorylation of p53 indicates the activation of p53 while phosphorylation at serine 46 phosphorylation generally leads to apoptosis. FACS analysis also showed increased amount of apoptosis 36 h post STZ treatment. Collectively, our results showed that STZ treatment leads to upregulation of GAD65 expression ultimately leading to apoptosis. We further wanted to see the kinetics of binding of SMAR1 and p53 to GAD65 promoter upon STZ treatment. ChIP analysis showed that there is a time dependent increase in association of SMAR1 and p53 on GAD65 promoter. Markers for chromatin activation AcH3K9 also showed increased amplification. The increase in recruitment of p300 to GAD65 promoter indicates chromatin activation that is reflected in increased GAD65 expression. On the other hand HDAC1 as well as Me3H4K20 showed time dependent loss of binding to GAD65 promoter. All these results indicate that upon STZ treatment, SMAR1 and p53 binds to GAD65 promoter and recruits p300 which leads to activation of GAD65 promoter. We further verified our results using mouse β-islets. Our results showed that STZ mediated upregulation of GAD65 expression is dependent on conflated action of p53 and SMAR1.

Interestingly, the analysis of promoter sequences of GAD65 across species from Human, mouse, rat and found that SMAR1 bound sequence is rather conserved amongst these species. Further alignment of mouse GAD65 promoter sequences with guinea pig, monkey, Bos Taraus and chimpanzee genomes showed that there is a great degree of conservation amongst all these species. The detailed alignment study is described in Additional file 2.

Thus our study for the first time shows the involvement of a MAR binding protein SMAR1 in regulation of GAD65 expression. p53 is already known to bind to and regulate expression of GAD67 isoform of GAD. In the present study we show that p53 also regulates expression of GAD65. Upon STZ treatment SMAR1 and p53 leads to upregulation of GAD65 expression which may be secreted in the blood. This circulating GAD65 is considered as an early marker for β-cell injury [37]. Taken together, our results show that GAD65 acts as one of the major injury marker as well as immuno-modulator. Thus regulation of GAD65 expression in β-islets by SMAR1 and p53 presents another facet of their involvement in DNA damage response.

## Conclusions

The present study delineates yet another facet of regulation of GAD65 expression by two tumor suppressor proteins SMAR1 and p53 synergistically. The study describes a novel pull down assay as a method to build library of DNA fragments bound to a protein *in-vitro*. By employing various techniques like ChIP, south western assays to study kinetics of DNA-protein interactions, we show that SMAR1 and p53 binds to GAD65 promoter in close vicinity to each other and upregulate GAD65 expression in a time and dose dependent manner upon STZ treatment. Induction of stress to β-islets by streptozotocin causing activates p53 and SMAR1 which inturn bind to GAD65 promoter and upregulating its expression. This increased production of GAD65 protein may be secreted in blood and detected as injury/stress marker to β-islets during onset of diabetes.

## Methods

### In-vitro DNA pull down assay and sequence alignment

This assay is designed to use recombinant proteins to elucidate DNA fragments associated with the protein. GST-SMAR1 recombinant protein was purified as per the previously described protocol [49]. The purity of recombinant protein was validated using 8% SDS-PAGE followed by silver stain. Mouse genomic DNA was sheared using Sonicator to obtain small fragments of approximately 500 bp. The purity and length of DNA was verified by running on 1.2% agarose gel. Just before the pull down reaction, the frozen recombinant protein was ultracentrifuged at 1, 00,000 rpm at 4°C to remove all the debris and unwanted protein aggregates. The DNA fragments were then incubated with glutathione bead bound GST-SMAR1 recombinant protein in DPD buffer (20 mM HEPES pH7.9, 1 mM EDTA, 30 mM KCl, 100 mM NaCl, 1 mM DTT, 25% Glycerol and 0.2% Tween-20) for 1 hrs at room temperature. The beads were than washed thrice with same buffer but increasing concentration of NaCl (100, 200, 300 mM NaCl) and bound DNA fragments were separated using phenol chloroform extraction. The fragments were than cloned into pGEMT easy vector and were subsequently sequenced using T7 specific primer. Glutathione beads as well as bead bound GST only were the controls used to rule out the non specific DNA binding of the recombinant protein. The resulting sequences were than aligned using MEME software and a 50mer consensus was derived.

### Cell culture and treatments

For in-vitro assays, RINm5F cell line was provided by NCCS cell repository. RINm5F cells were cultured in RPMI medium with 10% FCS in CO2 incubator. 1 μg of SMAR1, p53 or pTU puro SMAR1 (SiRNA) DNA was used for transfection per well using lipofectamine. P53 siRNA was procured from Santa Cruz and was used as per manufacturer’s instructions. For in-vivo experiments 4–6 weeks old C57b6, SMAR1 transgenic and p53 null mice were kindly provided by NCCS animal house facility. The animal studies were conducted in accordance with Principles of Laboratory Animal Care. STZ was reconstituted in chilled phosphate citrate buffer, pH 4.5 and immediately used for treatments.

### Immunoblotting and antibodies

Cells were scraped in 1XPBS and collected at indicated time points. Cells were then lysed using TNN buffer and protein concentration estimated using Bradford reagent (Bio-Rad). Equal amounts of proteins were taken for immunoblotting. Following sodium dodecyl sulphate (SDS)–polyacrylamide gel electrophoresis, the resolved proteins were electro blotted onto PVDF membrane (Amersham). The membrane was blocked overnight in Tris-buffered saline containing 0.1% Tween-20 (TBST) and 10% bovine serum albumin (BSA). The membrane was then probed with primary antibody in TBST for 2 h, followed by three 10-min TBST washes at room temperature. Incubation with the secondary antibody was done for 1 h, and three 10-min TBST washes were given prior to detection. Proteins were detected using enhanced chemiluminescence substrate (Amersham). The primary antibodies used were SMAR1 and S370 SMAR1 (Rabbit Polyclonal antibody raised in the lab; 49), p53 DO1 and actin (mouse monoclonal antibodies, Santa Cruz), ser15 p53 (#9284, Rabbit polyclonal; cell signaling), ser 46 p53(#2501, Rabbit Polyclonal; cell signaling), Ac 373/382 p53 (#2525, Rabbit Polyclonal; cell signaling) and GAD65 (mouse monoclonal antibody, #ab85866, Abcam). The secondary antibodies were donkey α-goat, donkey α-rabbit, donkey α-mouse (Bio-Rad).

### Purification of GST fusion protein

GST-SMAR1 as well as GST (160–350) and GST (350–548) truncation clones of SMAR1 are different truncations of total SMAR1 protein having DNA binding and protein interaction domains respectively [36,41]. All clones were grown in Luria-Bertani medium with ampicillin and induced with 1 mM isopropyl-D-thiogalactopyranoside (IPTG). Cells were resuspended in lysis buffer containing phosphate-buffered saline (PBS), Triton X-100, and protease inhibitors (Roche). After sonication and centrifugation, supernatant was incubated with glutathione Sepharose beads (Amersham) for 1 h at 4°C with gentle agitation. After three washes, with lysis buffer and PBS, the proteins were eluted at room temperature with 100 mM reduced glutathione buffer.

### Electrophoretic mobility shift assay (EMSA)

For EMSA, oligonucleotide labelling was done by a Klenow reaction using [α^32^P] dCTP in a 20 μl reaction containing 1 mM dATG mix, Klenow buffer, and 0.5U of Klenow DNA polymerase (Invitrogen). Probe purification was done using Probequant G-50 column (Amersham Biosciences). Binding reactions were performed in a 10 μl total volume containing 10 mM HEPES (pH 7.9), 1 mM dithiothreitol, 50 mM KCl, 2.5 mM MgCl_2_, 10% glycerol, 0.5 to 1 μg double-stranded poly (dI-dC), 10 μg BSA and 1 μg of recombinant protein. Samples were incubated for 5 min at room temperature prior to addition of radiolabeled probe. The samples were then incubated for 15 min at room temperature, and the products of binding reactions were resolved using 8% native polyacrylamide gel electrophoresis. The gels were dried under vacuum and processed for autoradiography.

### CNBr assay/Immunoaffinity purification

CNBr assay was done using previously described protocol by Nagore et al. [50]. GAD65 promoter and control DNA fragments were amplified by PCR followed be gel elution for purity. The fragments were than coupled to CNBr-activated sepharose beads (Stratagene) using coupling buffer (0.1 M NaHCO_3_, 0.5 M NaCl, pH 8.3). The cell lysate was then passed at a slow rate through the column after washes with equilibration buffer. Proteins were eluted using increasing concentrations of NaCl, pH 8.0 in 0.1 M Tris–glycine buffer pH 2.5. All elutes were pooled and concentrated followed by Western blot using SMAR1 antibody.

### Luciferase reporter assays

24 hours after transfection, the culture medium was removed, cells were washed with phosphate buffered saline (PBS), resuspended in 100 μl of cell lysis buffer. After freeze-thawing twice, lysed cells were spun at 9500 rpm for 15 min. Equal amount of protein was used for the assays. Luciferase activity was measured with Luclite substrate (Perkin Elmer, USA) and assay reactions read using Top-Count luminometer (Packard Life sciences, USA). Graphs were plotted from data obtained as a mean of 3 independent experiments along with computed standard deviations as error bars. The sequence of GAD65 promoter fragment used for luciferase assay is given in supplementary Figure 1.

### ChIP analysis

Assays were performed using Chromatin Immunoprecipitation (ChIP) assay kit (Upstate Biotechnology) following manufacturer’s instructions. 1x10^6^ cells were plated per 30 mm dish and treated with 3 mM STZ or left untreated. After treatment, DNA-protein interactions were fixed by adding formaldehyde directly to the media to a final concentration of 1%, incubated at 37°C for 10 min. Cells were washed with 1X PBS, pelletted and lysed in SDS-lysis buffer by sonication. The samples were then centrifuged at 15,000 rpm in order to clear the debris and chromatin extracts were incubated with 2 μg of the indicated antibody isotype control antibody and rotated at 4°C for 8–12 h. The antibody-chromatin complex was precipitated by adding protein A-sepharose bead, incubated for 4 h by rotating at 4°C and centrifuged at 3,000 rpm for 5 min. ChIP assays were carried out using SMAR1, HDAC1, acetyl Histone 3 Lysine-9 (H3K9), Histone 4 Lysine 16 (H4K16) and p53 antibodies (Cell Signaling). Input DNA, Rabbit IgG (r-IgG), and Mouse IgG (m-IgG) pulled DNA served as controls for all the experiments. Immunoprecipitated DNA was then subjected to 30 cycles of semi-quantitative PCR using the primers mentioned in Table 1.

**Table 1:** Primer sequences used for ChIP PCR analysis

**Primer Name**	**Sequence (5’-3’)**
**GAD 65 promoter (Fwd)**	**5’CTCCCTCTTTGGTTCCTTCC**
**GAD 65 promoter (Rev)**	**5’TGAGAGCTGTCTCTGGCTGA**
**GAD 67 promoter (Fwd)**	**5’CACCATGGGTAAGCCAGAC**
**GAD 67 promoter (Rev)**	**5’AGCACTTGTGGAGGAGCTG**
**Actin Promoter (Fwd)**	**5’GCCAGCAGCAAGCCTTGG**
**Actin Promoter (Rev)**	**5’GCCACTGGGCCTCCATTC**
**Consensus sequences (double stranded)**	**5’TAAAGTGTAAAGCCTGGGGTGCCTAATGAGATGAGCTAACTCACATTAATT**

### Cell cycle analysis by FACS

DNA content of cells was measured to check apoptosis. 1x 10^6^ RINm5F cells were seeded in a 6-well plate 24 hours before the day of experiment. RINm5F cells were treated with different concentrations of STZ 1, 2.5, 5 and 10 mM for 24 hours. The cells were fixed with cold 70% ethanol overnight and then treated with 10 μg/ml RNase at 37°C for 30 min. DNA content was measured by staining cells with propidium iodide (50 μg/ml) for 15 min. at RT. Apoptotic cells show low DNA stain ability resulting in a distinct, quantifiable region at sub-GO/G1 peak and analyzed by flow cytometry at 620 nm.

### β-islet isolation

Pancreas was dissected out from the mice and minced with scissor. Then 1–2 washings were given with plain DMEM. The minced tissue was let to settle for some time and supernatant was removed carefully. In a conical flask islet dissociation medium containing collagenase (Roche) and soybean trypsin inhibitor (Sigma-Aldrich) was added on the tissue and the mixture was stirred for 20 minutes. An ice cold RPMI with 10% FCS was added to stop the reaction. Mixture was centrifuged at low speed and the pellet was suspended in plain RPMI. The suspension was vortexed to detach acinar cells from islets and centrifuged again. Now the pellet was suspended in RPMI and floating islets were carefully removed by hand picking under microscope and proceeded for further treatments. The purity of the islets was confirmed using insulin staining.

### RT-PCR

Total RNA from RINm5F and islets was isolated by Tri-reagent (Sigma-Aldrich) according to manufacturer’s protocol. In brief, RINm5F cells or islets were lysed in Tri reagent by pipeting several times and mixed with chloroform and centrifuged at 12,000 g for 15 minutes. The aqueous phase was removed and RNA was precipitated in isopropanol. The RNA was pelletted at 10,000 g for 10 minutes, washed with ethanol and centrifuged. The pellet was air dried and suspended in DEPC water. cDNA was synthesized using 5 μg RNA as template in presence of MMLV-RT, MMLV-RT buffer, 10 mM dNTPs, DTT and RNAse OUT (All reagents were purchased independently from Invitrogen).

## Competing interests

The authors declare that they have no competing interests.

## Authors’ contributions

SS, VR, PP and SC have designed all the experiments. SS, TD, PLC, SK and VR have performed and analyzed all the experiments. Manuscript has been prepared by SS, PLC and SC. All authors read and approved the final manuscript.

## Supplementary Material

Additional file 1 Regulation of GAD65 expression by SMAR1 and p53 upon Streptozotocin treatment. Click here for file

Additional file 2 Regulation of GAD65 expression by SMAR1 and p53 upon Streptozotocin treatment. Click here for file
