# Correction to: Regulation of GAD65 expression by SMAR1 and p53 upon Streptozotocin treatment

**DOI:** 10.1186/s12860-020-00300-2

**Published:** 2020-08-17

**Authors:** Sandeep Singh, Varsheish Raina, Pavithra Lakshminarsimhan Chavali, Taronish Dubash, Sreenath Kadreppa, Pradeep Parab, Samit Chattopadhyay

**Affiliations:** 1 Samit Chattopadhyay, PhD, Scientist-G, National Centre for Cell Sciences, Pune, 411007 India; 2 Sandeep Singh, Assistant Professor, Centre for Human Genetics, School of Health Sciences, Central University of Punjab, Bathinda, 151001 India

**Correction to: BMC Molecular Biol 13, 28 (2012)**

**https://doi.org/10.1186/1471-2199-13-28**

Following publication of the original article [1], the authors reported that in Fig. 3C, the western blot data for GAD65 and p53 are the same.

Below is the correct Fig. 3C.

**Fig. 3:**
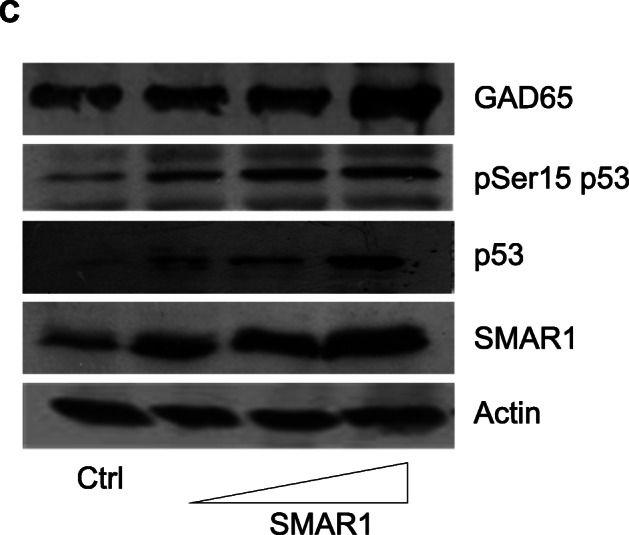
**SMAR1 upregulates GAD65 expression. C**. SMAR1 was over-expressed in Rin5f cells and samples were processed for western blot analysis 48 hrs post transfection. Figure shows western blot analysis of these samples using GAD65, phospho serine 15 p53 and SMAR1 expression while actin was used as loading control
