# Supplementary material for: Regulation of GAD65 expression by SMAR1 and p53 upon Streptozotocin treatment
Source: BMC Mol Biol. 2012 Sep 14;13:28. doi: 10.1186/1471-2199-13-28 (PMC3459802; doi:10.1186/1471-2199-13-28)
Supplement: Additional file 1 — Regulation of GAD65 expression by SMAR1 and p53 upon Streptozotocin treatment. [file 1471-2199-13-28-S1.docx]

**Supplementary file - 1**

**Regulation of GAD65 expression by SMAR1 and p53 upon Streptozotocin treatment**

*Sandeep Singh, Varsheish Raina, Sreenath Kadreppa, Pavithra Lakshminarsimhan Chavali, Taronish Dubash, Pradeep Parab and Samit Chattopadhyay*

**Map of transcription factor binding sites at GAD65 promoter in**

**vicinity of SMAR1 and p53 binding sites**

**GAD65 promoter**

**Analysis of binding sites in GAD65 promoter**

TGTCAGCTCTCCGCTGCTCCCTTTCAAGAA**GTTTCT**GTTCGTTTTATTTA    -951 **GATA1**

TTTAATTTTTCCCAGCCTGAGGTCCTCAGTGATAGACTCCAGCGTGGATT    -901

T**TAATTGC**TTCAATCAGCAGTCTTTCTCCTCAGCCGTCAGTCAAAACCTG    -851 **SMAR1** GACGGTGGGTCTGGGGACTCAGCGCTTGACT**GCACAGGA**AGTGGACGACC    -801 **p300**

CCCCCAGGCTGGCTCAGCTCAGTAGGCAGACGTTTTTAGGCTCGGCTAAG    -751

GAAGGAGATGAAATGAGTCCGTCTGCGTGGAAGCGATACACCTTCCTCCC    -701

TCTTTGGTTCCTTCCCCGTGCTCTGGAGCTCTGCCTGATTGCGGCCAGGT    -651

GGCCTGGGCTCTTGAGGTCACAGCGACCTCCGCACGGGTTTGGAGGAAGG    -601

AGGGTGCGAAAACCGCTTTTGCTCGGCCGGCCTCAGCCAG**AGACAGCTCT**    -551 **p53**

CAAACAATGAGCCAATCGCTGCACATGTGAAGTCGCTTTCAGTCACCCCC    -501

CAAGATTGTCA**AGGTCTCC**CTGGTGCCCAGGGTCACCGAGGCCAGCGTGC    -451 **GATA1**

AGCGGGCTGCGGTAGCACCCTGGACAGCGCGTCCCGGCCGAAGAGGACCT    -401

GGAGCGCCTGTGGCTGGGTCAGCGCTGCGCAGGCACGCCTCCCACCCCCG    -351

CATCTGACTCGCGCTCGAACGCACAGCCTCGCACCTCACGACCCAGCTCC    -301

TCCATTTCCCTTTCGTCACTCTCTGGTCTCCCCACCAAACTCCTCTCCCT    -251

AAAAATTCTCTTGGGTCCTTTCCACTTTCCAATCTCCAGACTGCGAGGAT    -201

CAGGCTACTCCCCCATTTAACCACCGCGCAGAGCACAACGGCTCGTCCCT    -151

ACGCCCTGACTCGAACACTCACACGTACGAGCACTGGCATACGCAGACAG    -101

CACGTTTCCTGTCCCTGTGTGACACCCACCCTCGTCGCGCGGCTGCTCCA     -51

GCCCTCGCGCGGTGCCCTCCTCCCGCCACACACACTCGCACACGCACGCA      -1
