# Supplementary material for: Regulation of GAD65 expression by SMAR1 and p53 upon Streptozotocin treatment
Source: BMC Mol Biol. 2012 Sep 14;13:28. doi: 10.1186/1471-2199-13-28 (PMC3459802; doi:10.1186/1471-2199-13-28)
Supplement: Additional file 2 — Regulation of GAD65 expression by SMAR1 and p53 upon Streptozotocin treatment. [file 1471-2199-13-28-S2.pdf]

## Supplementary file - 2

# Regulation of GAD65 expression by SMAR1 and p53 upon Streptozotocin treatment

*Sandeep Singh, Varsheish Raina, Sreenath Kadreppa, Pavithra Lakshminarsimhan Chavali, Taronish Dubash, Pradeep Parab and Samit Chattopadhyay*

**Sequence alignment of SMAR1 bound region of Gad65 promoter from mouse to study its evolutionary conservation**

### Rat (Promoter)

Score = 181 bits (200), Expect = 1e-42
Identities = 100/100 (100%), Gaps = 0/100 (0%)
Strand=Plus/Minus

```
Query  1         TTTAATTTTTCCCAGCCTGAGGTCCTCAGTGATAGACTCCAGCGTGGATTTTAATTGCTT
                 ||||||||||||||||||||||||||||||||||||||||||||||||||||||||||||
Rat              TTTAATTTTTCCCAGCCTGAGGTCCTCAGTGATAGACTCCAGCGTGGATTTTAATTGCTT

Query  61        CAATCAGCAGTCTTTCTCCTCAGCCGTCAGTCAAAACCTG   100
                 ||||||||||||||||||||||||||||||||||||||||
Rat              CAATCAGCAGTCTTTCTCCTCAGCCGTCAGTCAAAACCTG
```

### Mouse (Promoter)

Score = 163 bits (180), Expect = 3e-37
Identities = 96/100 (96%), Gaps = 0/100 (0%)
Strand=Plus/Plus

```
Query  1         TTTAATTTTTCCCAGCCTGAGGTCCTCAGTGATAGACTCCAGCGTGGATTTTAATTGCTT
                 ||||||||||| |||||||||||||||||||||||| |||||||||||||||||||||||
Mouse            TTTAATTTTTCTCAGCCTGAGGTCCTCAGTGATAGATTCCAGCGTGGATTTTAATTGCTT

Query  61        CAATCAGCAGTCTTTCTCCTCAGCCGTCAGTCAAAACCTG  100
                 |||||||||| ||||||| |||||||||||||||||||||
Mouse            CAATCAGCAGCCTTTCTCTTCAGCCGTCAGTCAAAACCTG
```

### Human (Promoter)

Score =  120 bits (132),  Expect = 4e-24
 Identities = 84/95 (88%), Gaps = 3/95 (3%)
 Strand=Plus/Plus

```
Query  6          TTTTTCCCAGCCTGAGGTCCTCAGTGATAGACTCCAGCGTGGATTTTAATTGCTTCAATC
                  |||| ||||||| ||||||||||||   ||||||||||||||||||||||||| ||||||
Human             TTTTCCCCAGCCCGAGGTCCTCAGT---AGACTCCAGCGTGGATTTTAATTGCCTCAATC

Query  66         AGCAGTCTTTCTCCTCAGCCGTCAGTCAAAACCTG  100
                  ||||||| |||||| ||||||||| ||| | ||||
Human             AGCAGTCATTCTCCCCAGCCGTCACTCAGAGCCTG
```

**Guinea Pig (putative Promoter)**

Score =  120 bits (132),  Expect = 4e-24
 Identities = 84/96 (88%), Gaps = 0/96 (0%)
 Strand=Plus/Minus

```
Query  5        ATTTTTCCCAGCCTGAGGTCCTCAGTGATAGACTCCAGCGTGGATTTTAATTGCTTCAAT
                ||| | ||| ||| ||||||||| |||| ||||||||||||||||||||||||| |||||
Pig             ATTCTCCCCGGCCCGAGGTCCTCGGTGAAAGACTCCAGCGTGGATTTTAATTGCCTCAAT

Query  65       CAGCAGTCTTTCTCCTCAGCCGTCAGTCAAAACCTG  100
                ||||||| || |||| ||||||||| ||| ||||||
Pig             CAGCAGTGTTCCTCCCCAGCCGTCAATCATAACCTG
```

**Bos Taurus (Whole genome alignment)**

Score =  118 bits (130),  Expect = 1e-23
 Identities = 80/90 (89%), Gaps = 0/90 (0%)
 Strand=Plus/Plus

```
Query  11         CCCAGCCTGAGGTCCTCAGTGATAGACTCCAGCGTGGATTTTAATTGCTTCAATCAGCAG
                  ||||||||||||| ||  |||||||| ||||||||| ||||||||||| |||||||||||
Bos Taurus        CCCAGCCTGAGGTTCTTGGTGATAGATTCCAGCGTGAATTTTAATTGCCTCAATCAGCAG


Query  71         TCTTTCTCCTCAGCCGTCAGTCAAAACCTG  100
                  |||||||||  |||||||||||| ||| ||
Bos Taurus        TCTTTCTCCCAAGCCGTCAGTCAGAACTTG
```

**Chimpanzee (Whole genome alignment)**

Score =  111 bits (122),  Expect = 2e-21

Identities = 82/95 (86%), Gaps = 3/95 (3%)
Strand=Plus/Plus

```
Query  6         TTTTTCCCAGCCTGAGGTCCTCAGTGATAGACTCCAGCGTGGATTTTAATTGCTTCAATC
                 |||| ||||||| ||||||||| ||   ||||||||||||||||||||||||| ||||||
Chimpanzee       TTTTCCCCAGCCCGAGGTCCTCGGT---AGACTCCAGCGTGGATTTTAATTGCCTCAATC

Query  66        AGCAGTCTTTCTCCTCAGCCGTCAGTCAAAACCTG  100
                 ||||||| |||||| |||| |||| ||| | ||||
Chimpanzee       AGCAGTCATTCTCCCCAGCTGTCACTCAGAGCCTG
```

**Rhesus monkey (Whole genome alignment)**

Score = 105 bits (116), Expect = 8e-20
Identities = 81/95 (85%), Gaps = 7/95 (7%)
Strand=Plus/Plus

```
Query  1         TTTAATTTTTCCC--AGCCTGAGGTCCTCAGTGATAGACTCCAGCGTGGATTTTAATTGC
                 |||| ||||||||  |||| |||||||||     ||||||||||||||||||||||||||
Monkey            TTTATTTTTTCCCCCAGCCCGAGGTCCTC-----TAGACTCCAGCGTGGATTTTAATTGC

Query  59        TTCAATCAGCAGTCTTTCTCCTCAGCCGTCAGTCA  93
                  ||||||||||||| |||| | ||||||||| |||
Monkey            CTCAATCAGCAGTCATTCTTCCCAGCCGTCACTCA
```
